# Food Production Index Forecasting for Sustainable Food Systems in Türkiye: A Machine Learning-Based Approach

**DOI:** 10.3390/foods15162814

**Published:** 2026-08-12

**Authors:** Ferhan Balci Torun, Mehmet Kayakuş, Onder Kabas, Georgiana Moiceanu, Mariana-Gabriela Munteanu

**Affiliations:** 1 Department of Gastronomy and Culinary Arts, Faculty of Tourism, Akdeniz University, 07058 Antalya, Türkiye; ferhanbalci@akdeniz.edu.tr; 2 Department of Biomedical Engineering, Faculty of Engineering, Akdeniz University, 07058 Antalya, Türkiye; mehmetkayakus@akdeniz.edu.tr; 3 Department of Machine, Technical Science Vocational School, Akdeniz University, 07070 Antalya, Türkiye; 4 Department of Entrepreneurship and Management, Faculty of Entrepreneurship, Business Engineering and Management, National University of Science and Technology Politehnica Bucharest, 060042 Bucharest, Romania; georgiana.moiceanu@upb.ro; 5 Biotechnical Systems Department, Faculty of Biotechnical Systems Engineering, National University of Science and Technology Politehnica Bucharest, 060042 Bucharest, Romania

**Keywords:** sustainable food systems, food production index, food security, machine learning, food system resilience, resource efficiency, data-driven policymaking, sustainable agriculture

## Abstract

Sustainable food systems are increasingly challenged by climate change, resource constraints, market volatility, and growing food demand, making accurate forecasting of food production essential for food security and long-term sustainability. Despite the growing use of machine learning in agricultural forecasting, studies directly modeling the Food Production Index (FPI) within a sustainable food systems framework remain limited, particularly in emerging economies. This study addresses this gap by forecasting Türkiye’s Food Production Index using agricultural, macroeconomic, and trade-related indicators covering the period 1962–2023. Seven predictive approaches, including Multiple Linear Regression (MLR), Bayesian Ridge Regression, Support Vector Regression (SVR), Random Forest, Gradient Boosting, Artificial Neural Networks (ANNs), and K-Nearest Neighbors (KNN), were comparatively evaluated using R^2^, RMSE, and MAE metrics. The results demonstrate that Bayesian Ridge Regression (R^2^ = 0.968) and MLR (R^2^ = 0.918) significantly outperform more complex machine learning algorithms, indicating that model–data compatibility is more critical than algorithmic complexity in long-term food production forecasting. The findings reveal that economic growth, agricultural inputs, and structural transformation processes play a decisive role in shaping food production dynamics. By integrating machine learning with sustainability-oriented food system analysis, this study provides a robust evidence base for supporting food security strategies, resource-efficient agricultural planning, and resilient food system governance. The proposed framework offers macro-level decision-support insights for policymakers engaged in long-term food system planning, strategic risk monitoring, and evidence-based policy evaluation.

## 1. Introduction

Food production is regarded as a fundamental system situated at the intersection of food security, economic growth, and sustainable development goals. Globally, a growing population, climate change, and resource constraints necessitate a comprehensive analysis of food production systems—not only in terms of production capacity but also regarding their environmental and social impacts [1]. In this context, the Food Production Index (FPI) is one of the most important measures of how well countries are doing in terms of agricultural production compared to a certain reference period. It also gives a way to compare food supply capacity [2,3]. Developed by the Food and Agriculture Organization and the World Bank, this index serves as a crucial tool representing the “availability” dimension of food security and is at the center of policy discussions directly linked to Sustainable Development Goals such as SDG 2 (End Hunger), SDG 12 (Responsible Consumption and Production), and SDG 13 (Climate Action) [3].

As increasingly emphasized in the literature, food production is not a one-dimensional output variable but rather a multi-layered system shaped by the interaction of economic, environmental, and social factors [4]. Studies conducted recently have shown that increases in food production can exacerbate environmental pressures. It is estimated that the food system’s contribution to global greenhouse gas emissions accounts for between 21% and 37% of total human-induced emissions; this figure is known to stem from agricultural land use, livestock activities, and energy consumption in production processes [5]. Analyses specific to Türkiye reveal that agricultural production, livestock farming, and food production increase the ecological footprint, while certain sub-sectors, such as aquaculture, may have a mitigating effect on this pressure [6]. However, it is important to note that the overall impact of these activities on the environment can vary significantly based on practices and technologies used in each sub-sector. This finding raises the question of whether the FPI calculation methodology fully reflects holistic ecological pressures and indicates that the index should be considered alongside multidimensional variables in sustainability analyses.

A study that comparatively examined the effects of food production on sustainable development at the sectoral level found, based on Panel ARDL results for 23 upper-middle-income countries, that food production increases CO_2_ emissions in the long term; conversely, it revealed a statistically significant and positive relationship with life expectancy at birth. The fact that Türkiye is included in this group of countries is particularly significant, as it provides a direct basis for comparison in the current analysis. The observation of positive effects on life expectancy alongside the positive relationship between food production and carbon emissions indicates that this system exhibits a two-way structure in terms of sustainable development and that this structure must be addressed beyond traditional one-dimensional analyses [7].

The findings reveal that the relationship between food production and sustainable development is not linear or unidirectional; rather, it involves a complex balance between environmental costs and social benefits. Studies in the literature that examine the FPI’s relationship not only with production capacity but also with food security, nutrition, and well-being indicators emphasize that this index must be analyzed within a multidimensional framework [8].

When examined specifically in the context of Türkiye, it is evident that the dynamics of agricultural production are closely linked to economic growth, foreign trade, and structural transformation processes. Türkiye has undergone a unique structural transformation process in which the agricultural sector’s share of GDP has declined significantly over the decades, while the absolute size of value added and the volume of exports have continued to increase [9]. It has been demonstrated that the increase in agricultural production and agricultural exports creates positive effects on economic growth; this relationship involves bidirectional causality and directly shapes the country’s policy needs regarding rural development and sustainable food security [10]. However, while the agricultural sector’s share in the economy has decreased, its production structure has evolved into a more efficient and technology-intensive model, and labor intensity has declined alongside increased mechanization, which has led to higher productivity and competitiveness in both domestic and international markets. Agricultural production is shaped by the interaction of capital, land use, foreign trade, and technological advancements, and this situation demonstrates that food production dynamics require a multidimensional analysis. From the perspective of sustainable food systems, it is evident that Türkiye has made some progress on relevant SDG indicators within the framework of national policy documents and the transformation of the agri-food sector; however, no meaningful progress has been achieved on indicators related to food and nutrition over the past decade, and deficiencies in monitoring and evaluation mechanisms have limited policy effectiveness [11]. In this context, the sustainability of the food supply chain emerges as a critical area that must be addressed beyond mere production capacity indicators; studies on Türkiye’s food sector reveal that a multi-parameter evaluation framework encompassing customer satisfaction, product safety, and resource use efficiency serves as a complementary component to FPI-focused analyses [12].

Similarly, the international literature emphasizes the strong relationships between food production and economic growth, natural resource use, and technological advancements. In this context, food production is viewed not only as an economic output but also as a critical area of analysis in terms of environmental sustainability and policymaking [1].

In recent years, sustainable food systems have emerged as a strategic priority for policymakers and researchers due to increasing concerns regarding climate change, resource scarcity, food losses, and supply chain disruptions. Ensuring long-term food security requires not only increasing agricultural productivity but also improving the efficiency, resilience, and sustainability of food production systems. Consequently, data-driven analytical frameworks and machine learning approaches are increasingly being integrated into food system research to support evidence-based decision making and enhance the sustainability of food supply chains. Within this perspective, the Food Production Index can be considered a valuable indicator for monitoring the performance and resilience of national food systems.

A review of the literature on modeling food production dynamics reveals that traditional econometric and time-series methods have dominated the field for many years. Researchers widely use approaches such as ARDL, VAR, FMOLS, and similar methods to analyze long-term relationships between food production and related variables. In addition to econometric approaches, traditional forecasting models such as Autoregressive Integrated Moving Average (ARIMA), Seasonal ARIMA (SARIMA), and exponential smoothing have been widely used in agricultural and economic forecasting due to their ability to capture temporal dependence, seasonality, and trend structures [13]. ARIMA-based models are particularly effective for univariate time-series forecasting when strong autocorrelation patterns exist, while exponential smoothing methods are often preferred for stable trend extrapolation under limited data conditions. However, these conventional approaches mainly rely on linear assumptions and may be limited in capturing complex multivariate interactions among agricultural, macroeconomic, and trade-related variables. In contrast, machine learning approaches offer greater flexibility in modeling nonlinear and high-dimensional relationships, although their superiority depends strongly on data structure, sample size, and feature complexity [14]. Accordingly, machine learning models were not assumed to universally outperform traditional forecasting approaches; rather, they were included to examine whether additional predictive gains could be achieved when modeling multidimensional interactions beyond conventional univariate time-series structures. However, these methods largely rely on linear assumptions and may be limited in handling complex data structures [15], which can lead to inaccurate predictions and a failure to capture the nonlinear relationships that often exist in food production dynamics. In recent years, with the advancement of data-driven approaches, machine learning methods have emerged as a significant alternative for forecasting food production. Artificial neural networks, regression-based algorithms, and ensemble methods can deliver high performance, particularly in modeling multivariate and nonlinear relationships [16,17]. As a unique contribution to national-level food production forecasting, Nosratabadi et al. (2021) conducted a study comparing ANFIS and MLP algorithms; they demonstrated that ANFIS achieved the lowest error value using generalized bell-shaped membership functions, thereby showing that such models could serve as a reliable decision-support tool for policymakers [17]. A review of the broader ML literature on agricultural yield and production forecasting reveals that methods such as Random Forest and Support Vector Regression consistently demonstrate success in crop classification and yield estimation applications, and since these studies hold the potential to strengthen policy interventions in the context of food security and environmental sustainability, they are also linked to the SDG literature [18,19,20]. Hybrid and AI-focused architectural approaches remain at the forefront of efforts to improve prediction accuracy; it is emphasized that AI-supported prediction models have the potential to transform food supply chain optimization and regional agricultural decision-making processes [21].

Recent studies further highlight the growing importance of artificial intelligence in agricultural forecasting and sustainable food systems. Dhal and Kar (2024) demonstrated that AI-driven forecasting models can substantially improve agricultural productivity prediction while enhancing food supply chain optimization and food security planning [21]. Similarly, Herteux et al. (2024) showed that real-time data integration significantly improves food security forecasting by enabling faster and more adaptive decision-making [22]. At the crop-production level, Mishra et al. (2024) reported that predictive analytics can support sustainable agricultural planning and market resilience through improved production forecasting [23]. More recently, Ali et al. (2025) emphasized that artificial intelligence technologies are becoming central to sustainable agriculture by improving productivity, resource efficiency, and resilience against environmental uncertainty [24]. These recent developments further support the relevance of machine learning-based forecasting frameworks for analyzing long-term food production dynamics.

The selection of predictor variables in this study is grounded in a structured agricultural economic framework reflecting key transmission mechanisms within sustainable food systems. Agricultural input variables, including fertilizer consumption, agricultural land, and arable land, directly affect production capacity by influencing yield and land productivity. Macroeconomic indicators such as GDP, inflation, and official exchange rate influence food production indirectly through capital investment, production costs, and input affordability. Exchange rate fluctuations are highly relevant in Türkiye due to their impact on imported agricultural inputs such as fertilizers, machinery, and energy-related logistics costs. Structural and trade-related variables, including rural population, agricultural value added, and food exports/imports, reflect labor availability, sectoral productivity, market competitiveness, and external dependency. Together, these variable groups provide a multidimensional representation of the economic, structural, and production-related determinants shaping long-term Food Production Index dynamics. To evaluate both linear and potential nonlinear relationships among these determinants, the study employs both conventional statistical models and machine learning algorithms.

However, it is noteworthy that findings regarding the performance of these methods in the literature are inconsistent. While some studies demonstrate that more complex models exhibit superior performance, others show that simpler, linear models produce more balanced and reliable results by better adapting to the data structure [24]. This situation highlights that model performance does not depend solely on algorithmic complexity; rather, factors such as the structure of the dataset, relationships between variables, and sample characteristics are decisive. As highlighted by Wolpert & Macready’s (2002) “No Free Lunch” theorem, no learning algorithm can universally outperform others across the entire problem space; this finding underscores the need to evaluate model selection separately for each dataset [25]. Studies indicate that in macroeconomic and aggregated datasets, a limited number of observations increases the risk of overfitting in high-parameter models, and simpler approaches—such as Bayesian regularization mechanisms—can yield more successful results in this context. Recent studies in the Turkish context on predicting sustainability indicators using machine learning also support this general pattern, demonstrating that simpler models often outperform more complex ones when dealing with limited data, particularly in macroeconomic applications. Kayakuş and Akkaya (2026) modeled Türkiye’s national sustainability performance using multiple ML algorithms, revealing that technical capacity, economic structure, social development, and environmental conditions play a decisive role; the study provides a direct methodological foundation for how a sector-specific FPI-focused framework can be linked to multidimensional sustainability analyses [26].

When all these findings are evaluated together, it becomes evident that the Food Production Index has a multidimensional structure and is strongly interrelated with economic, environmental, and social systems. However, there is a notable inconsistency in the literature regarding the performance of the methods used to model these relationships. The majority of existing studies in the literature focus on yield estimation at the product level or on the total agricultural production index; however, Türkiye-focused studies that directly estimate the FPI using machine learning, relate it to long-term macroeconomic variables, and integrate it into a sustainable development framework represent a significant gap in the literature [16,17]. In this context, this study aims to evaluate food production dynamics through a sustainable food systems perspective and to investigate how machine learning-based forecasting models can support food security, resource efficiency, and evidence-based policymaking in Türkiye. In addition, more accurate forecasting of national food production may contribute to maintaining stable raw material availability for the food industry, strengthening food supply chain resilience, and supporting proactive food safety planning by enabling earlier responses to potential production shortages.

It should be noted that the sustainability perspective adopted in this study primarily reflects macro-level economic and production resilience rather than full environmental sustainability. Due to long-term data availability constraints, environmental indicators such as climate variability, water availability, land degradation, biodiversity, and carbon emissions were not incorporated into the empirical model. Therefore, the present framework should be interpreted as a partial representation of sustainable food systems, with future studies encouraged to integrate environmental sustainability dimensions for more comprehensive modeling. Despite this limitation, the selected indicators remain theoretically relevant because economic resilience, production capacity, and trade adaptability constitute core structural dimensions of food system sustainability at the national level.

This study offers four major contributions to the literature. First, it provides the first long-term machine learning-based forecasting framework for Türkiye’s Food Production Index using annual data spanning 1962–2023. Second, it integrates FPI forecasting with a sustainable food systems perspective by jointly modeling agricultural, macroeconomic, and trade-related variables. Third, it systematically compares linear and nonlinear machine learning models under identical analytical conditions. Finally, the findings demonstrate that simpler regularized models such as Bayesian Ridge Regression may outperform more complex nonlinear models in small-sample macroeconomic datasets.

From a theoretical perspective, this study conceptualizes food production dynamics through four major transmission mechanisms within sustainable food systems. First, agricultural input factors directly influence production capacity through productivity enhancement. Second, macroeconomic variables affect food production indirectly through capital accumulation, inflationary pressure, and input affordability. Third, trade-related factors influence food system resilience by altering import dependency and export competitiveness. Finally, long-term structural transformation processes reshape labor allocation, production efficiency, and rural agricultural capacity. By integrating these interacting mechanisms into a unified analytical framework, this study contributes to the existing literature by extending Food Production Index forecasting within a sustainable food systems perspective.

## 2. Materials and Methods

**Figure 1:**
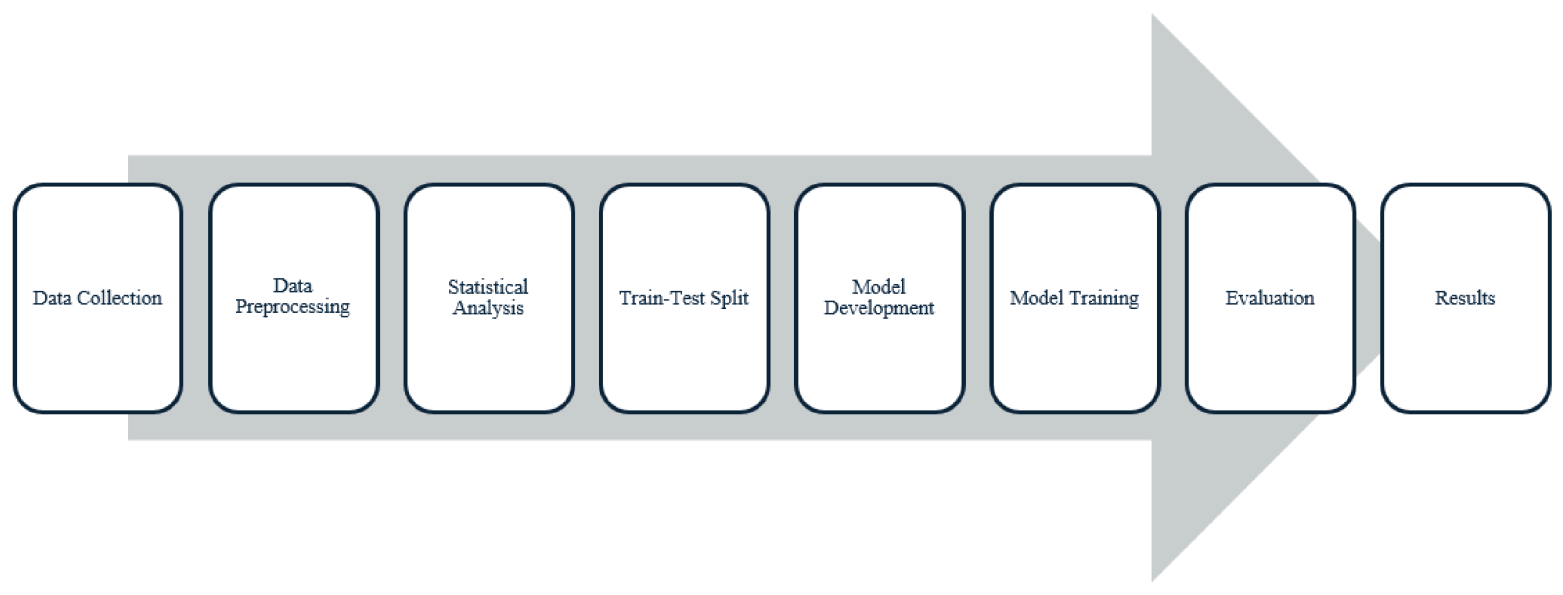
Flowchart illustrates the overall methodological framework of the study, including data collection, preprocessing, machine learning model development, validation, and performance evaluation.

To understand the dynamics of food production, agricultural, macroeconomic, and trade-based variables were analyzed together; the relationships among these variables were evaluated within a multidimensional framework. Systematic data preprocessing steps were applied during the analysis process; subsequently, models were developed using both traditional statistical methods and various machine learning algorithms. Appropriate validation approaches were used to evaluate model performance in an objective and comparable manner. The data preparation, modeling, and evaluation steps were designed as a sequential, integrated process, and the overall methodological flow of the study is presented in the flowchart shown in Figure 1.

### 2.1. Data Set

The dataset used in this study was obtained from the World Bank Open Data database due to its reliability and the provision of long-term, consistent data [27]. The primary reason for including Türkiye in the analysis is that the country has undergone a transition over the past sixty years from an agriculture-dominated economy to one in which the industrial and service sectors play a more dominant role. The dataset covering the 1962–2023 period allows for the continuous tracking of this transformation process and enables the simultaneous assessment of both long-term structural changes and cyclical economic fluctuations affecting food production. In this regard, the dataset provides a suitable foundation for analyzing the economic and structural dimensions of food production dynamics, though it does not capture the environmental dimension of sustainability, which is addressed separately below.

The Food Production Index was used as the dependent variable in the analysis. Considering that food production is shaped not only by agricultural inputs but also by economic and commercial factors [28], the independent variables were categorized into three main groups in line with the literature.

- Agricultural Input Factors:
  ○ Fertilizer consumption (kg/hectare);
  ○ Agricultural land (% of total land);
  ○ Arable land (% of total land).
- Macroeconomic Indicators:
  ○ GDP (billion USD, constant 2015);
  ○ Inflation (consumer prices) (annual %, consumer prices);
  ○ Official exchange rate (local currency/US dollar);
  ○ Trade (% of GDP) (% of gross domestic product).
- Structural and Foreign Trade Indicators:
  ○ Rural population (% of total population);
  ○ Agriculture value added (% of gross domestic product);
  ○ Food Exports (% of merchandise exports);
  ○ Food Imports (% of merchandise imports).

**Figure 2:**
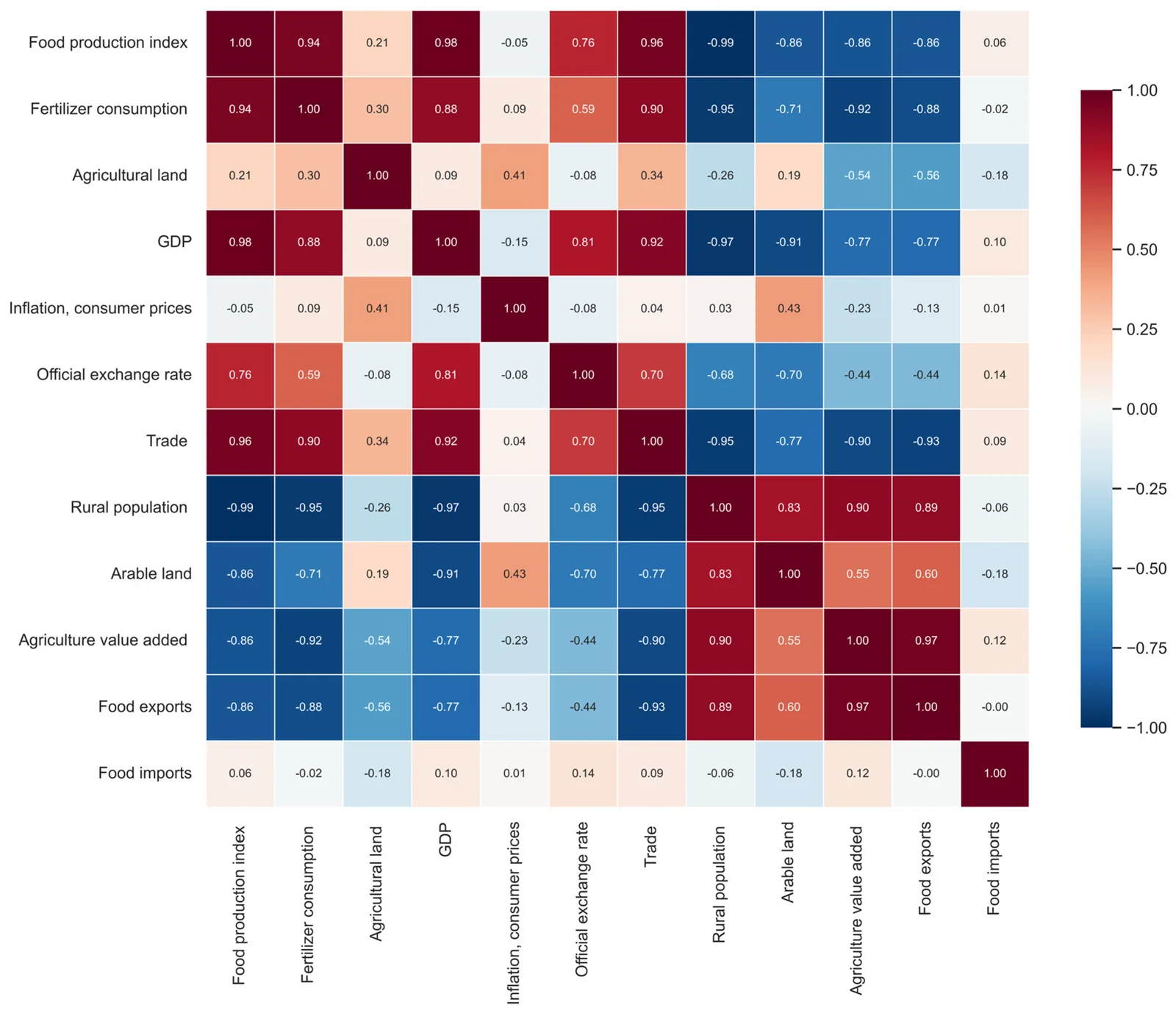
Correlation matrix of the Food Production Index and candidate variables.

This dataset aims to provide a broader economic analysis by considering that food production is not solely dependent on agricultural inputs but is also shaped by changes in the economic structure, trade dynamics, and population structure; it does not, however, constitute a comprehensive assessment of environmental sustainability. The correlation matrix, which reveals the relational structure among the variables in the dataset, is presented in Figure 2.

The correlation matrix presented in Figure 2 illustrates the linear relationships among the candidate variables initially considered in the study. The findings indicate the presence of high levels of both positive and negative correlations among certain variables. It was found that variables representing economic growth—Gross Domestic Product (GDP), trade openness, fertilizer consumption, and exchange rate—exhibit strong positive relationships with the Food Production Index. This suggests that economic development, integration into international trade, and increased use of agricultural inputs are positively associated with food production.

In contrast, it was observed that rural population, arable land, agriculture value added, and food exports exhibit strong negative relationships with the Food Production Index. This finding indicates an inverse association between these structural changes and food production dynamics during the study period. It also points to structural changes in agricultural production and the transition to more efficient production techniques.

Feature selection in this study was not based solely on correlation coefficients. Instead, a multi-criteria selection strategy combining correlation analysis, Variance Inflation Factor (VIF), and domain knowledge was adopted. Correlation analysis was used only as an exploration step to understand pairwise relationships among variables. Final variable inclusion was determined by considering multicollinearity, theoretical relevance to food production dynamics, and predictive contribution within the machine learning framework. This approach enabled the construction of a statistically robust and interpretable predictor set while reducing the risk of excluding potentially important variables. Agricultural Land, Inflation, and Food Imports showed relatively weak correlations with the Food Production Index and were therefore not retained as contemporaneous predictors for subsequent predictive modeling. The remaining candidate predictors and their one-period lagged counterparts were subsequently evaluated for multicollinearity using VIF analysis.

Multicollinearity analyses supported the findings, as correlation analysis results alone are insufficient for variable selection. Since highly correlated predictors may introduce multicollinearity and destabilize coefficient estimation, additional statistical assessment using Variance Inflation Factor (VIF) analysis was performed. Variables with VIF values above the commonly accepted threshold of 5 were carefully examined for redundancy and theoretical relevance. Rather than relying solely on mechanical exclusion, final predictor selection considered both statistical diagnostics and agricultural economic interpretability to preserve meaningful explanatory variables within the modeling framework. Table 1 presents descriptive statistics for the candidate variables initially considered in the study. These variables were evaluated during the preliminary correlation analysis to examine their relationships with the Food Production Index. Agricultural Land, Inflation, and Food Imports were included in this initial assessment and are therefore reported in Table 1 for completeness; however, they were not retained as predictors in the subsequent predictive modeling stage because of their relatively weak correlations with the Food Production Index. During the iterative VIF screening process, highly collinear variables were sequentially removed until the predefined threshold was satisfied. The variables excluded during the overall predictor-selection process included both contemporaneous and one-period lagged predictors. During the preliminary correlation screening, Agricultural Land, Inflation, and Food Imports were not retained as contemporaneous predictors because of their relatively weak correlations with the Food Production Index. During the subsequent VIF screening of the remaining candidate predictors, the excluded contemporaneous predictors were GDP, Rural Population, Agriculture Value Added, Arable Land, Food Exports, and Trade. Several one-period lagged predictors were also excluded during VIF screening, including Rural Population (lag-1), Food Production Index (lag-1), Agriculture Value Added (lag-1), Trade (lag-1), GDP (lag-1), Official Exchange Rate (lag-1), Fertilizer Consumption (lag-1), Food Exports (lag-1), Arable Land (lag-1), Agricultural Land (lag-1), and Inflation (lag-1). The remaining predictors satisfied the predefined multicollinearity criterion and were subsequently used for model development.

VIF analysis revealed severe multicollinearity before screening, with several predictors substantially exceeding the conventional threshold (VIF > 5), and the maximum VIF reaching 1437.61 for lagged rural population. After iterative VIF-based screening, all retained predictors exhibited acceptable multicollinearity levels (VIF < 2), substantially improving coefficient stability and model interpretability. The final predictor set used in both Bayesian Ridge Regression and Multiple Linear Regression (MLR) therefore consisted of Fertilizer Consumption and Official Exchange Rate, both included as contemporaneous predictors. The same predictor set was used for both models to ensure direct comparability.

Table 1 presents descriptive statistics for the candidate variables initially considered in the study and provides an overview of their distributional characteristics. The Food Production Index has a mean value of 64.52 and ranges from 28.12 to 128.92, indicating substantial variation over the study period. Fertilizer Consumption also exhibits considerable variability, reflecting changes in agricultural input use over time. Agricultural Land and Arable Land show relatively limited variation, whereas GDP, Inflation, the Official Exchange Rate, and Trade exhibit greater variability over the study period. The variation observed in Rural Population reflects the long-term demographic transformation and urbanization process in Türkiye, while Agriculture Value Added, Food Exports, and Food Imports display varying degrees of change associated with structural and trade-related dynamics. Agricultural Land, Inflation, and Food Imports were included in the preliminary correlation assessment but were not retained as predictors in the subsequent predictive modeling stage because of their relatively weak correlations with the Food Production Index. Following this preliminary screening, the remaining candidate predictors were further assessed for multicollinearity using Variance Inflation Factor (VIF) analysis, and the final predictor set was determined based on the predefined selection criteria. Overall, the descriptive statistics illustrate the differing distributional characteristics of the agricultural, macroeconomic, demographic, and trade-related indicators initially considered in the study.

#### Stationarity Diagnostics

To evaluate the time-series properties of the dataset, stationarity diagnostics were performed using the Augmented Dickey–Fuller (ADF) test prior to model development. The results of the ADF test are presented in Table 2. The findings indicate that most variables, including the Food Production Index, were non-stationary at level form (*p* > 0.05), reflecting strong long-term trends in agricultural and macroeconomic indicators. Only Agriculture Value Added and Food Imports exhibited stationarity at conventional significance levels. These findings suggest that the dataset contains substantial trend components. However, because the primary objective of this study is predictive forecasting rather than causal inference or parameter estimation, non-stationary variables were retained in their original form to preserve long-term structural information relevant for forecasting. This approach is consistent with predictive modeling studies in macroeconomic forecasting, where preserving trend information may improve long-horizon predictive performance.

These findings suggest that the dataset contains substantial trend components. However, because the primary objective of this study is predictive forecasting rather than causal inference or parameter estimation, non-stationary variables were retained in their original form to preserve long-term structural information relevant for forecasting. This approach is consistent with predictive modeling studies in macroeconomic forecasting, where preserving trend information may improve long-horizon predictive performance.

### 2.2. Machine Learning

Machine learning is a subfield of artificial intelligence that uses algorithms capable of automatically learning patterns and relationships from data [29]. Unlike traditional statistical methods, it relies on a data-driven learning process rather than rigid, predefined assumptions [30]. As a result, it achieves high success, particularly in modeling complex, nonlinear, and multivariate relationships [31]. Machine learning methods are widely used to improve prediction accuracy and uncover hidden relationships among variables in fields such as economic, financial, and agricultural analysis.

All analyses were conducted using Python 3.11 within a Jupyter Notebook (version 7.6.1) environment. The primary libraries used were pandas (2.2), NumPy (1.26), scikit-learn (1.5), matplotlib (3.9), and seaborn (0.13). To ensure reproducibility, a fixed random seed (random_state = 42) was used in all stochastic algorithms. All models were implemented using a standardized machine learning pipeline consisting of preprocessing, scaling, training, validation, and performance evaluation stages.

The algorithms used in the modeling process were configured using commonly adopted parameter settings reported in the literature to ensure fair comparability across models. Parameter values were selected based on prior studies and adjusted to reflect the structural characteristics of the dataset.

For nonlinear machine learning models, parameter configurations commonly reported in the prior literature were adopted to ensure fair comparability across algorithms. Parameters were selected based on previous empirical studies and adjusted according to the structural characteristics of the dataset, including limited sample size and temporal dependency. This configuration-based approach enabled a balanced comparison among nonlinear models without relying on exhaustive hyperparameter optimization. Hyperparameter configurations for nonlinear models were selected using literature-guided constrained parameter settings rather than exhaustive optimization, primarily to reduce overfitting risk under the small-sample setting. For SVR, kernel type, regularization strength (C), and epsilon sensitivity were varied within commonly reported ranges. For Random Forest and Gradient Boosting, tree depth, number of estimators, and learning rate parameters were adjusted conservatively to avoid excessive model complexity. For KNN, multiple neighborhood sizes were evaluated. Despite these adjustments, nonlinear models remained highly sensitive to the limited sample size, strong multicollinearity, and temporal dependence. The candidate hyperparameter values were evaluated through a limited manual search based on previous empirical studies and preliminary experiments. Final configurations were selected according to predictive performance while prioritizing model stability and minimizing overfitting under the relatively small sample size.

The final model configurations used in the analyses were as follows. SVR was implemented with a radial basis function (RBF) kernel (C = 100, ε = 0.1). Random Forest was configured with 300 trees (n_estimators = 300), a maximum tree depth of 3 (max_depth = 3), and a minimum leaf size of 3 (min_samples_leaf = 3). Gradient Boosting used 100 estimators (n_estimators = 100), a learning rate of 0.05, and a maximum tree depth of 2. ANN was implemented using an MLPRegressor with a single hidden layer containing 10 neurons, ReLU activation, L2 regularization (α = 0.01), and a maximum of 3000 training iterations. KNN was implemented with k = 3 nearest neighbors. Bayesian Ridge Regression and Multiple Linear Regression were implemented using the default scikit-learn parameter settings.

#### 2.2.1. Multiple Linear Regression (MLR)

Multiple linear regression (MLR) is one of the most fundamental and widely used statistical methods for modeling the linear relationship between a dependent variable and multiple independent variables. In this model, the dependent variable is expressed as a linear combination of the independent variables, and the effect of each variable is measured through regression coefficients [32]. The MLR method is based on the assumptions that error terms are normally distributed, there is no multicollinearity among the variables, and the relationship is linear. Therefore, the model’s interpretability is quite high, providing direct information about the direction and magnitude of the variables [33]. However, its predictive performance may be limited in datasets with a high prevalence of nonlinear relationships. In this study, MLR was used as a baseline comparison model to evaluate the performance of other machine learning methods.

#### 2.2.2. Bayesian Ridge Regression

Bayesian Ridge Regression is an approach that frames regression analysis within a probabilistic framework, in which parameters are represented by probability distributions rather than fixed values [34]. In this method, prior distributions are defined for model parameters, and posterior distributions are obtained by combining these prior distributions with observational data [35]. The Bayesian Ridge Regression approach offers significant advantages in terms of explicitly expressing model uncertainty and providing more flexible results with small sample sizes. Additionally, expressing parameter estimates as probability distributions rather than confidence intervals offers a more comprehensive interpretability. However, the challenges of determining appropriate prior distributions and the high computational costs are among the method’s limitations [36]. In this study, Bayesian Ridge Regression was evaluated for its ability to manage uncertainty and provide an alternative modeling approach.

#### 2.2.3. Support Vector Regression (SVR)

Support vector regression (SVR) is a powerful machine learning method based on support vector machines that can model both linear and nonlinear relationships. SVR aims to determine the optimal regression function by ignoring prediction errors that fall within a specified error tolerance (epsilon). This approach improves generalization performance by preventing the model from overfitting [37]. Kernel functions (e.g., RBF, polynomial) enable the transformation of data into higher-dimensional spaces, effectively capturing complex relationships [38]. SVR stands out for providing high prediction accuracy, particularly in small and medium-sized datasets. However, the selection of model parameters (C, epsilon, kernel parameters) must be done carefully. In this study, SVR was used as an effective method for modeling nonlinear relationships.

#### 2.2.4. Random Forest

Random forest is one of the ensembles learning methods created by combining many decision trees. In this method, each decision tree is trained using subsamples and subsets of variables randomly selected from the dataset. The final prediction is obtained by averaging the output of all the trees. This approach significantly improves generalization performance while reducing the risk of overfitting [39]. Random Forest also supports model interpretability by providing the ability to determine variable importance levels. It is a powerful method for capturing nonlinear relationships and demonstrating effective performance on high-dimensional datasets [40]. In this study, Random Forest was used both to assess prediction accuracy and to determine the relative importance of variables.

#### 2.2.5. Gradient Boosting

Gradient boosting is an ensemble learning method that builds a powerful model by sequentially training weak learners. In this approach, each new model is trained to minimize the errors of the previous model, thereby gradually reducing the total error. The gradient boosting algorithm uses the gradient descent principle to minimize the loss function [41]. This method provides very high prediction accuracy, particularly in complex and nonlinear data structures. However, there is a risk of overfitting, and hyperparameters such as the learning rate and tree depth must be carefully tuned [42]. In this study, gradient boosting has been incorporated into the model as an advanced method that delivers strong predictive performance.

#### 2.2.6. Artificial Neural Networks (ANNs)

Artificial neural networks (ANNs) are a machine learning method developed by drawing inspiration from the neural structure of the human brain, capable of learning complex data relationships through multi-layered structures [43]. ANNs consist of input, hidden, and output layers, and the connections between the neurons in each layer are represented by weights. The learning process is carried out by updating these weights using the backpropagation algorithm. ANNs produce highly successful results with nonlinear and high-dimensional datasets. However, it is important to have sufficient data during model training and to set the hyperparameters correctly, as inadequate data or poorly chosen hyperparameters can lead to overfitting or underfitting, ultimately affecting the model’s performance [44]. In this study, ANNs were used to model complex relationships and achieve high prediction accuracy.

#### 2.2.7. K-Nearest Neighbors (KNN)

K-nearest neighbors (KNN) is a non-parametric and relatively simple machine learning method that operates based on the similarity between samples [45]. In this method, a prediction for an observation is determined based on the values of the K nearest neighboring observations [46]. The KNN algorithm makes no assumptions about data distribution and therefore offers a flexible structure. However, as the dataset grows, the computational cost increases, and the model’s performance varies depending on the distance metric and the K parameter used. Additionally, distance calculations between variables can be misleading if no scaling is performed, as unscaled features can disproportionately influence the distance metrics, leading to inaccurate results in the KNN algorithm [47]. In this study, KNN was used to compare it with other methods and to provide a simple reference model.

## 3. Results

This study compares the performance of machine learning models developed to forecast the Food Production Index in Türkiye. During the analysis, one-period lag (lag-1) values were generated for all numerical variables, considering the time-dependent nature of the dataset, thereby creating an extended data structure that incorporates information from previous periods. Throughout the manuscript, variables reported without the “lag-1” designation refer to contemporaneous predictors, whereas variables identified as “lag-1” represent their corresponding one-period lagged values. This approach was adopted to enhance the model’s predictive performance in dynamic processes, such as agricultural production, that are influenced by past values. After generating the one-period lagged variables, missing observations resulting from lag construction were removed, and both contemporaneous and lagged candidate predictors were subsequently evaluated during the predictor-selection process [47].

Initially, a chronological 70:30 train–test split was applied, corresponding to approximately 43 training observations and 18 testing observations after lag construction. Given the temporal structure of the dataset, observations were split sequentially without random shuffling to preserve chronological order and reduce data leakage. In addition to the conventional train–test split, an expanding-window walk-forward validation strategy was implemented to provide a more rigorous evaluation of forecasting robustness. In this approach, models were iteratively trained using all available historical observations and subsequently evaluated on the next unseen observation. This validation procedure preserves temporal order, minimizes data leakage, and provides a more realistic assessment of predictive performance under real-world forecasting conditions [47].

During the data preprocessing stage, mean imputation was used to minimize the impact of missing values. In addition, standardization (StandardScaler) was applied to eliminate the negative impact of scale differences between variables on model performance, particularly in algorithms with high scale sensitivity (SVR, KNN, artificial neural networks, and linear models) [48]. With this method, all variables were transformed so that their mean was zero and their standard deviation was one, ensuring consistent model training.

Seven different machine learning methods were used in the study to capture both linear and non-linear relationships: Multiple linear regression (MLR), Bayesian Ridge Regression, support vector regression (SVR), random forest, gradient boosting, artificial neural networks (ANNs), and K-nearest neighbors (KNN). Furthermore, correlation analysis and multicollinearity assessments were considered during the variable selection process. Model stability was improved by evaluating predictors using correlation structure, Variance Inflation Factor (VIF), and theoretical relevance to food production dynamics, while leveraging the inherent robustness of certain algorithms (particularly Bayesian approaches and tree-based methods) against multicollinearity.

Three key performance metrics—the coefficient of determination (R^2^), root mean square error (RMSE) and mean absolute error (MAE)—were used to evaluate model performance. These metrics were chosen because they allow for the assessment of model performance from different perspectives. The R^2^ provides information about overall fit by indicating the extent to which the model explains the variance in the dependent variable [48]. RMSE allows for the evaluation of the model’s performance in the face of significant deviations by assigning greater weight to large errors. MAE, on the other hand, treats all errors equally, offering a simpler and more understandable measure of the average prediction error. The combined use of these three metrics ensures a more comprehensive analysis of both the models’ overall explanatory power and the distribution of errors [49]. The findings reveal significant differences among the various algorithms in terms of prediction accuracy and error levels, indicating that some algorithms outperform others in specific contexts, such as handling outliers or providing consistent predictions across different datasets.

Although alternative parameter configurations were considered during model development, Bayesian Ridge Regression and Multiple Linear Regression remained the most reliable approaches in terms of predictive accuracy and generalization performance. The performance results for all models are presented in Table 3.

The findings presented in Table 3 highlight substantial differences in predictive performance across the evaluated machine learning algorithms. The results indicate that linear models exhibit significantly superior performance on macroeconomic time series data compared to more complex, nonlinear models. This suggests that model success is largely related to the data structure and the algorithms’ generalization capacity.

The Bayesian Ridge Regression (R^2^ = 0.968) and MLR (R^2^ = 0.918) models achieved the highest prediction accuracy. This finding suggests that the relationship between the Food Production Index and the selected predictors is predominantly linear. In particular, the Bayesian Ridge Regression model is seen to effectively manage the multicollinearity problem identified in previous analyses through its regularization mechanism. Furthermore, the limited number of observations covering the 1962–2023 period (approximately 62 observations) allowed simpler and parametric models to produce more stable and reliable predictions without the risk of overfitting. In contrast, the ANN model demonstrated moderate performance with an R^2^ value of 0.731; although it possesses the capacity to model nonlinear relationships, it exhibited lower predictive success compared to linear models due to the limited size of the current dataset and its sensitivity to hyperparameters.

The initially low and negative R^2^ values observed for SVR, Random Forest, Gradient Boosting, and KNN suggest that these algorithms faced challenges in adapting to the current data structure. However, this performance should not be interpreted solely as an inherent limitation of these algorithms.

Negative R^2^ indicates that the model performs worse than simply predicting the mean of the test set. This outcome suggests that highly flexible nonlinear algorithms overfit the training subset while failing to generalize to unseen temporal observations.

Model performance in nonlinear machine learning methods is highly sensitive to hyperparameter selection, model complexity, and data representation. Therefore, part of the observed performance degradation may be attributable to suboptimal parameter configurations, in addition to the limited sample size and strong linear structure of the dataset.

Accordingly, the negative R^2^ values should not be interpreted as evidence that these algorithms are inherently unsuitable for food production forecasting, but rather as an indication that the combination of limited sample size, temporal dependence, and the fixed hold-out evaluation constrained their generalization performance in the present application.

**Figure 3:**
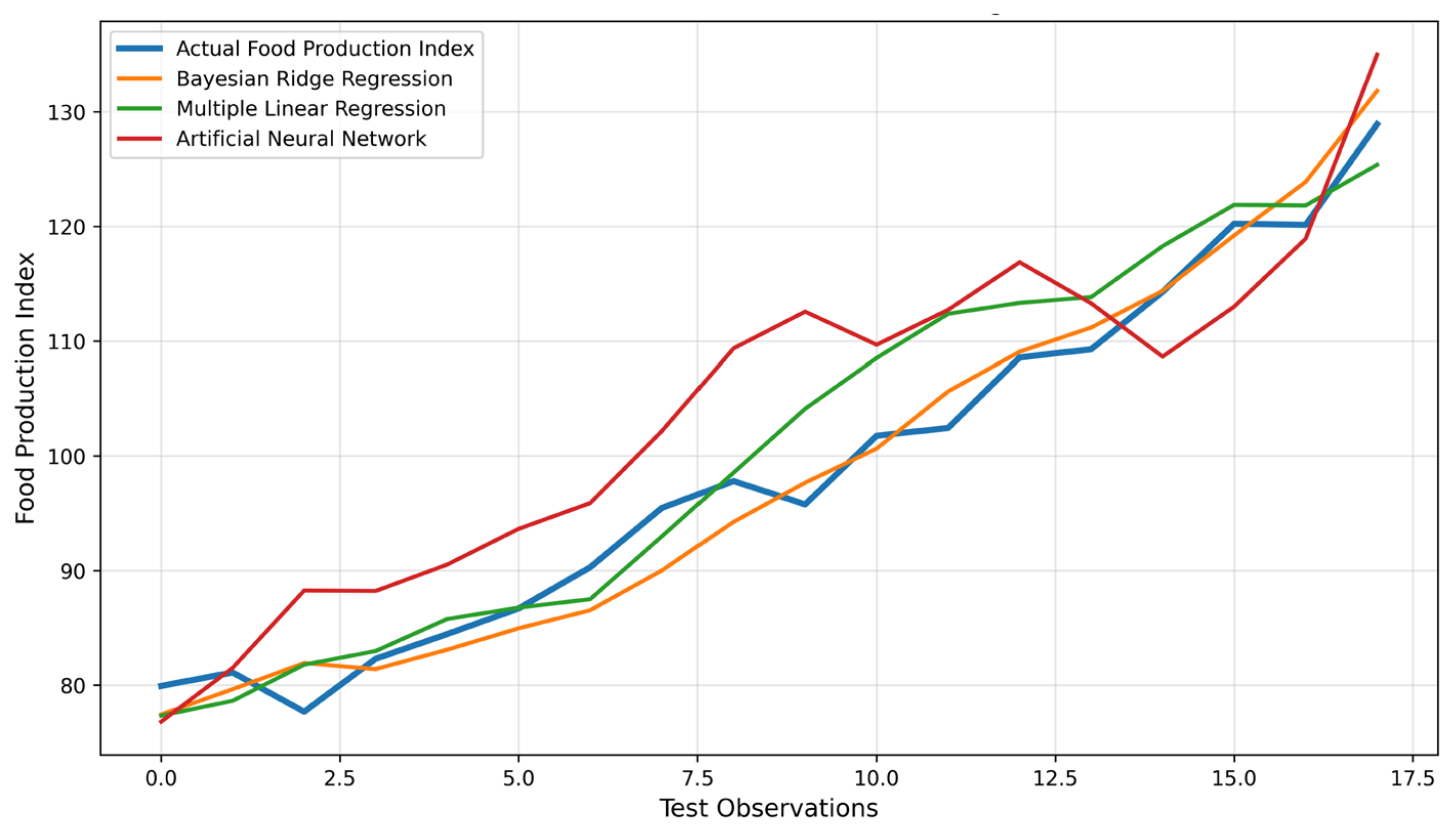
Comparative prediction error plot showing deviations between predicted and actual Food Production Index values for the best-performing machine learning models.

In this context, it is also important to visually evaluate model performance not only through numerical error metrics but also through the distribution of prediction errors and the extent to which models can track actual values. Accordingly, the prediction results of the three models showing the highest performance were examined comparatively. The prediction error plot presented in Figure 3 illustrates how well these models capture the true values and the behavior of the error distributions over time.

Figure 3 provides a comparative analysis of the forecast performance of different models on the Food Production Index. Upon examining the graph, it is evident that only Bayesian Ridge Regression and Multiple Linear Regression successfully capture the overall trend and remain closely aligned with the actual Food Production Index values. However, some differences in forecasting accuracy among the models are notable. The results show that the Bayesian Ridge Regression and MLR models produce results that are quite close to the actual values and make forecasts with low error, particularly for intermediate observations. In contrast, the ANN model shows greater deviation from actual values at certain points, particularly exhibiting a tendency toward overestimation during specific periods. Nevertheless, the ANN model captures the overall trend but remains more sensitive to short-term fluctuations, leading to less reliable forecasts during volatile periods. These findings highlight that model selection should be evaluated not only in terms of capturing the general trend but also in terms of error distribution and stability.

**Figure 4:**
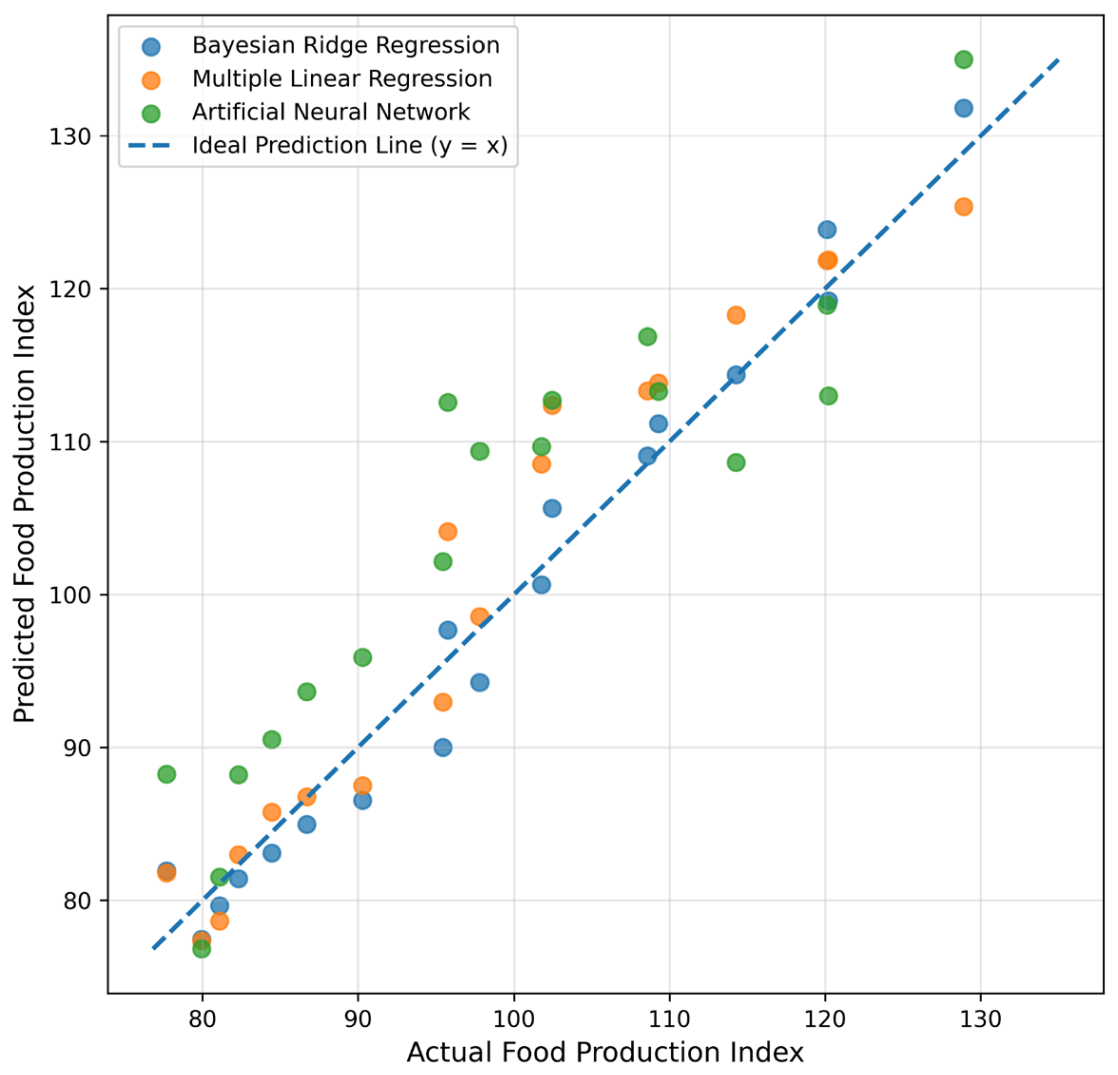
Scatter plot comparing predicted versus actual Food Production Index values. The dashed 45-degree reference line represents ideal prediction accuracy.

The relationship between model predictions and actual values is shown in the scatter plot presented in Figure 4.

The scatter plot presented in Figure 4 illustrates the relationship between the values predicted by the models and the actual values. The dashed reference line (the 45-degree line) in the graph represents the ideal situation where the predictions are exactly equal to the actual values. The proximity of the points to this line is evaluated as an indicator of the model’s prediction accuracy. The results indicate that the predictions from the Bayesian Ridge Regression and MLR models are generally distributed closer to the reference line and thus exhibit higher accuracy. In contrast, it is notable that the points associated with the ANN model are spread over a wider area and deviate significantly from the reference line in some observations. This indicates that the ANN model produces higher prediction errors at certain data points and has higher variance. The findings indicate that reliable trend capture is primarily limited to Bayesian Ridge Regression and Multiple Linear Regression, whereas nonlinear models exhibit substantially weaker predictive alignment. Overall, it is concluded that the Bayesian Ridge Regression and MLR models offer more stable and reliable prediction performance, while the ANN model exhibits a tendency toward over- or under-prediction in some observations.

**Figure 5:**
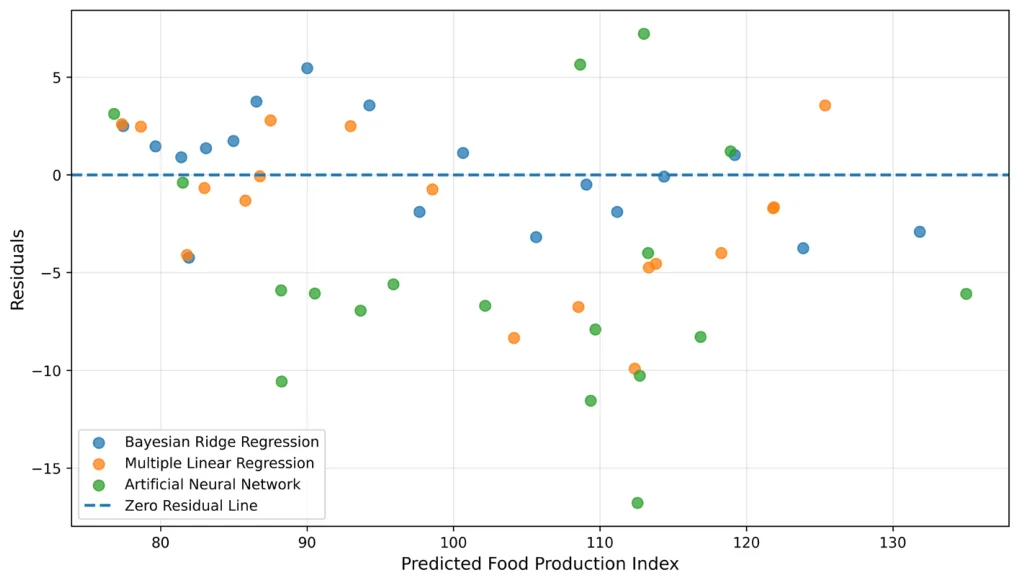
Residual plot illustrating prediction errors across observations for the best-performing models, used to assess error distribution, variance, and model stability.

The error structure of the models and the distribution of residuals are visualized in the residual plot shown in Figure 5.

The residual plot presented in Figure 5 provides valuable insights when evaluating the distribution of prediction errors and model performance. The residual plot shows that around the zero line in the plot, it can be observed that the errors associated with the Bayesian Ridge Regression and MLR models are generally distributed more evenly and randomly around zero. This indicates that these models do not produce systematic errors and that their predictions are more consistent. In contrast, it is notable that the residuals from the ANN model are distributed over a wider range and contain larger deviations, particularly in the negative direction. This suggests that the ANN model tends to underestimate the true values in some observations and has higher error variance. Additionally, the fact that residuals exhibit a specific pattern in certain regions may indicate that the model fails to fully capture the entire data structure. Overall, it is concluded that the Bayesian Ridge Regression and MLR models exhibit a more balanced and stable error distribution, whereas the ANN model demonstrates higher variance and an irregular error structure.

To further evaluate temporal generalization performance, an expanding-window walk-forward validation was performed. The results of this validation procedure are presented in Table 4.

The walk-forward validation results presented in Table 4 further confirm the robustness of the proposed forecasting framework. Bayesian Ridge Regression maintained the highest predictive performance (R^2^ = 0.955), followed by Multiple Linear Regression (R^2^ = 0.905), indicating strong temporal generalization. Although several nonlinear models demonstrated improved performance under walk-forward validation compared with the conventional split approach, Bayesian Ridge Regression and linear models remained the most stable and accurate. The substantial performance improvement observed for several nonlinear models under walk-forward validation primarily results from the expanding-window evaluation strategy rather than major model modifications. Unlike the fixed hold-out split, walk-forward validation progressively enlarges the training set at each iteration, allowing nonlinear models to learn more stable temporal patterns and reducing variance. No major hyperparameter changes were introduced between the two evaluation settings. Bayesian and linear models remained the most stable and accurate throughout the validation process.

To further evaluate whether the proposed machine learning framework provides predictive advantages over conventional time-series forecasting techniques, benchmark comparisons were performed using ARIMA and Holt exponential smoothing models. The comparative benchmark results are presented in Table 5.

The benchmark comparison presented in Table 5 provides additional insight into the relative performance of machine learning and traditional forecasting approaches. Although ARIMA and Holt exponential smoothing demonstrated competitive predictive performance under walk-forward validation, Bayesian Ridge Regression remained the best-performing model. Holt Exponential Smoothing achieved a slightly lower MAE than ARIMA, which may be attributed to its ability to capture the relatively smooth long-term trend of the Food Production Index without introducing additional autoregressive complexity. However, the performance difference between the two traditional forecasting models was small. These findings suggest that while traditional univariate forecasting models effectively capture the trend structure of annual macroeconomic series, incorporating agricultural, macroeconomic, and trade-related explanatory variables through Bayesian Ridge Regression provides additional predictive value. This result supports the usefulness of multivariate forecasting frameworks beyond simple univariate trend extrapolation.

## 4. Discussion

The dataset used in this study provides a robust basis for analysis due to its long-term time series structure covering the period from 1962 to 2023, the absence of missing data, and the inclusion of both agricultural and macroeconomic variables. However, as revealed by correlation and VIF analyses, there is a high degree of multicollinearity among the variables. Furthermore, the dataset’s relatively limited number of observations increases the risk of overfitting, particularly for non-parametric and highly flexible machine learning models. The relatively limited effective sample size after lag construction (approximately 61 observations) substantially constrained the learning capacity of highly flexible nonlinear algorithms. However, the applied VIF-based screening reduced the risk of severe coefficient instability and improved overall model robustness. The relatively small effective sample size should also be interpreted in relation to model complexity. Highly flexible machine learning algorithms, including tree-based ensemble methods and artificial neural networks, generally require substantially larger datasets to estimate many parameters reliably and to achieve stable generalization performance. In contrast, regularized linear models are less data-demanding and are therefore more appropriate for small-sample forecasting problems. This interpretation is consistent with the established machine learning literature emphasizing that model complexity should be matched to data availability to minimize overfitting and improve predictive reliability [50,51,52,53].

Bayesian Ridge Regression achieved the strongest overall predictive performance. This finding can be interpreted considering broader methodological debates in forecasting research. In agricultural and macroeconomic forecasting, the relative success of a model depends not solely on algorithmic sophistication but on the compatibility between model assumptions and data structure. The present dataset exhibits three defining characteristics: limited sample size, strong multicollinearity, and pronounced long-term trends. Under such conditions, highly flexible nonlinear algorithms often suffer from unstable parameter estimation and reduced generalization capacity. In contrast, Bayesian Ridge Regression benefits from regularization, which stabilizes coefficient estimates and reduces overfitting by shrinking less informative parameters. This finding supports the argument that simpler regularized models may outperform more complex machine learning architectures when forecasting long-term macroeconomic series with limited observations. This interpretation is consistent with the broader forecasting literature, which suggests that increased algorithmic complexity does not necessarily guarantee superior predictive performance, particularly in structured time-series forecasting problems [50,54].

From a sustainable food systems perspective, these findings should be interpreted primarily at the macro level, with direct implications for strategic policy intervention. The results suggest that policymakers should prioritize monitoring Fertilizer Consumption and Official Exchange Rate, as these variables were retained as the final predictors and showed the strongest associations with long-term Food Production Index dynamics. From a policy perspective, fertilizer input conditions and exchange-rate volatility are particularly relevant because of their potential implications for agricultural production costs and input affordability. When the forecasting framework predicts a potential decline in food production, governments may respond through targeted fertilizer subsidies, strategic input support programs, trade-balancing measures, and food reserve management policies. In this context, the proposed framework can function as an early warning and decision-support tool, enabling proactive intervention before production shocks significantly affecting food security. Beyond agricultural policy, these forecasting capabilities are also relevant to food processing industries and supply chain managers. Earlier identification of production fluctuations may improve raw material planning, reduce supply disruptions that could compromise food quality, and support preventive food safety management throughout the agri-food supply chain.

The reported β coefficients were obtained after standardization of the predictor variables and should therefore be interpreted as relative measures of association and predictor importance rather than as coefficients expressed in the original measurement units. It is important to note that these standardized coefficients reflect statistical associations within the fitted models rather than causal effects; therefore, no causal claims regarding the relationships between the predictors and the Food Production Index should be inferred from these results. The standardized coefficient analysis indicated that Fertilizer Consumption showed the strongest positive association with the Food Production Index in both Bayesian Ridge Regression and Multiple Linear Regression (β ≈ +19.4), followed by Official Exchange Rate (β ≈ +8.2). These coefficients correspond to the predictors retained in the final model specification.

In contrast, the relatively lower performance of more complex machine learning methods such as SVR, Random Forest, Gradient Boosting, ANN, and KNN can be attributed to the structural characteristics of the dataset. The limited number of observations, high multicollinearity, and pronounced linear trends have constrained the flexibility advantage of these methods and, in some cases, led to overfitting. In particular, the high error variance and irregular residual distribution observed in the ANN model indicate that the model’s generalization ability remains limited. Similarly, tree-based methods failed to generate sufficient diversity due to high correlation in the dataset, while KNN was negatively affected by the data distribution due to its distance-based structure.

The findings indicate that datasets characterized by high correlation, limited observations, and pronounced temporal trends may favor simpler regularized models over highly flexible nonlinear algorithms. These results emphasize that forecasting methodology should be selected based on data characteristics rather than model complexity alone, particularly in agricultural and food systems research.

Although the study period (1962–2023) includes major economic crises, policy reforms, and structural transformations in Türkiye, the primary objective of this study was predictive forecasting rather than causal econometric inference. Therefore, formal structural break detection and cointegration analyses were not incorporated into the proposed machine learning framework. Instead, chronological data partitioning together with expanding-window walk-forward validation was adopted to evaluate model robustness under evolving temporal conditions while preserving the natural temporal ordering of the observations.

## 5. Conclusions

This study examines the dynamics of food production in Türkiye from a long-term perspective using the Food Production Index and presents a comprehensive model framework by jointly evaluating agricultural, macroeconomic, and trade-based variables. The findings demonstrate that reliable long-term food production forecasting depends on selecting models that align with the structural characteristics of the dataset. From a practical perspective, the proposed framework should primarily be interpreted as a macro-level analytical tool supporting long-term strategic planning, trend monitoring, and policy scenario evaluation in food system governance. Rather than providing direct guidance for localized operational interventions, such as region-specific subsidy allocation or precision farming decisions, the model offers broader insights into structural factors influencing national food production dynamics. Accordingly, the proposed framework mainly supports strategic agricultural planning and long-term food security assessment. Furthermore, improved long-term forecasting of food production may help maintain stable raw material availability for food manufacturing, strengthen the resilience of food supply chains, and support proactive food quality and food safety management by enabling earlier planning for potential production fluctuations. In line with current global priorities related to sustainable food systems and resource efficiency, the findings highlight the growing importance of advanced analytical and predictive approaches in shaping future food policies. From a sustainable development perspective, its ability to reveal how food production is shaped by economic growth, trade, and structural transformation processes provides significant contributions to the design of sustainable agricultural policies and the strengthening of food security. This study contributes by providing an integrated framework linking agricultural, macroeconomic, and trade-related determinants to long-term Food Production Index forecasting within a sustainable food systems perspective. From an applied perspective, the study offers an empirical contribution to the sustainability literature by illustrating, through data-driven modeling, how macro-level resilience, adaptive capacity, and structural stability accompany environmental indicators as relevant dimensions of food system sustainability. In this regard, the study contributes to the examination of the Food Production Index through data-driven modeling approaches, thereby introducing a methodological and practical dimension to the literature on sustainable development. The benchmark analysis further indicates that traditional forecasting models such as ARIMA and exponential smoothing remain useful for annual trend-dominated series; however, multivariate machine learning frameworks offer additional explanatory power by integrating structural drivers of food production. Nevertheless, the selected economic, agricultural, and trade-related indicators should be interpreted as representing important structural dimensions of sustainable food systems rather than providing a comprehensive assessment of environmental sustainability.

This study has several limitations that should be acknowledged. First, the dataset consists of only 62 annual observations, which restricts the learning capacity of data-intensive machine learning algorithms and may limit model generalization. Second, the annual temporal resolution may obscure short-term fluctuations, seasonal variability, and sudden structural changes affecting food production dynamics. Although Augmented Dickey–Fuller (ADF) diagnostics were incorporated to assess stationarity, formal cointegration testing and structural break detection were intentionally not included, as the primary objective of this study was predictive forecasting rather than causal econometric inference. Consequently, potential non-stationarity and undetected structural breaks over the 1962–2023 period—driven by major economic crises and policy reforms—may still influence model coefficients and forecasting stability and should be interpreted with appropriate caution. Future studies should explicitly test structural breaks and long-run cointegration relationships to strengthen the robustness of these findings. Third, important climatic and environmental variables such as precipitation, drought severity, temperature anomalies, and water availability could not be incorporated due to data limitations, potentially restricting the sustainability dimension of the analysis. As a result, the present framework should not be interpreted as a direct tool for environmental shock forecasting or localized resilience planning. Fourth, despite multicollinearity assessment, strong correlations among macroeconomic predictors may still influence coefficient stability and model sensitivity. Finally, since the analysis focuses exclusively on Türkiye, caution is required when generalizing the findings to other countries with different agricultural, economic, and climatic conditions. An additional limitation of the present study is the absence of explainable AI techniques such as SHAP or permutation importance, which could provide deeper insights into the relative contribution of predictors. Similarly, although Bayesian Ridge Regression provides probabilistic parameter estimation, posterior distributions and credible intervals were not reported because the primary objective of this study was comparative predictive forecasting rather than Bayesian parameter inference. Future studies may incorporate full posterior uncertainty estimates to further improve model interpretability. Future research should integrate explainability frameworks to better understand how individual variables influence Food Production Index forecasting and to improve model transparency for policy applications.

In future studies, the use of higher-frequency datasets or the creation of panel data structures could enhance model performance and enable more detailed analyses. Integrating indicators related to climate change, water resources, and environmental sustainability into the model will contribute to a more comprehensive assessment of the sustainability dimension of the Food Production Index. Additionally, comparing different modeling strategies using time-series-based deep learning models and hybrid approaches could improve prediction accuracy. Applying the proposed model framework to different countries or regional datasets constitutes an important area of research for testing the generalizability of the findings and contributing to sustainable development policies on a broader scale. Future studies may also incorporate benchmark forecasting approaches such as ARIMA, SARIMA, or Prophet to enable broader comparative evaluation.

## Figures and Tables

**Table 1:** Descriptive statistics of candidate variables.

	**Average**	**Minimum**	**Maximum**	**SD**	**SE**
Food Production Index	64.52	28.12	128.92	26.54	3.40
Fertilizer consumption	68.71	3.20	149.64	36.45	4.67
Agricultural land	50.40	47.62	53.56	1.48	0.19
GDP (billion USD, constant 2015)	404.16	74.11	1212.97	302.92	38.78
Inflation	33.01	1.12	105.21	28.58	3.66
Official exchange rate	1.24	0.00	16.55	2.65	0.34
Trade	34.60	8.33	79.90	18.06	2.31
Rural population	46.44	10.82	75.92	19.73	2.53
Arable land	30.46	25.44	33.49	2.41	0.31
Agriculture value added	20.25	5.70	53.33	14.12	1.81
Food exports	28.80	8.11	66.99	18.78	2.41
Food imports	4.93	0.87	13.82	2.47	0.32

**Table 2:** Augmented Dickey–Fuller stationarity test results.

Variable	ADF Statistic	*p*-Value	Result
Food Production Index	4.3179	1.0000	Non-stationary
Fertilizer Consumption	−0.7471	0.8342	Non-stationary
Agricultural Land	−2.0142	0.2804	Non-stationary
GDP	1.6914	0.9981	Non-stationary
Inflation	−1.9697	0.3000	Non-stationary
Official Exchange Rate	0.0097	0.9593	Non-stationary
Trade	0.8591	0.9925	Non-stationary
Rural Population	1.4356	0.9973	Non-stationary
Arable Land	0.0096	0.9593	Non-stationary
Agriculture Value Added	−3.5605	0.0066	Stationary
Food Exports	−2.3979	0.1423	Non-stationary
Food Imports	−3.3293	0.0136	Stationary

**Table 3:** Predictive performance of the evaluated machine learning models using the chronological 70:30 hold-out validation strategy.

Model	R^2^	RMSE	MAE
MLR	0.918	4.329	3.469
Bayesian Ridge Regression	0.968	2.695	2.294
SVR	−9.624	49.311	44.876
Random Forest	−2.873	29.774	25.571
Gradient Boosting	−2.658	28.935	24.647
ANN	0.731	7.845	6.903
KNN	−3.075	30.540	26.529

**Table 4:** Predictive performance of machine learning models under the expanding-window walk-forward validation strategy.

Model	R^2^	RMSE	MAE
MLR	0.904	4.672	3.672
Bayesian Ridge Regression	0.955	3.205	2.671
SVR	0.132	14.090	9.955
Random Forest	0.486	10.845	10.309
Gradient Boosting	0.830	6.229	5.559
ANN	0.802	6.724	5.331
KNN	0.825	6.312	5.801

Note: Expanding-window walk-forward validation sequentially enlarges the training set by incorporating all previously available observations before predicting the next unseen observation, thereby preserving temporal order and reducing data leakage.

**Table 5:** Benchmark comparison with traditional forecasting models under expanding-window walk-forward validation.

Model	R^2^	RMSE	MAE
Bayesian Ridge Regression	**0.955**	**3.205**	**2.671**
ARIMA (auto-AIC)	0.857	3.242	2.867
Holt Exponential Smoothing	0.858	3.180	2.796

## Data Availability

The original data were obtained from the World Bank Open Data database. The processed dataset supporting the findings of this study has been deposited in the Zenodo repository and is publicly available at https://doi.org/10.5281/zenodo.21227970. The Python scripts and model implementation details are available from the corresponding author upon reasonable request.

## Abbreviations

AI	Artificial Intelligence
ANN	Artificial Neural Network
ARDL	Autoregressive Distributed Lag
ARIMA	Autoregressive Integrated Moving Average
CO_2_	Carbon Dioxide
FAO	Food and Agriculture Organization
FMOLS	Fully Modified Ordinary Least Squares
FPI	Food Production Index
GDP	Gross Domestic Product
GB	Gradient Boosting
KNN	K-Nearest Neighbors
MAE	Mean Absolute Error
ML	Machine Learning
MLR	Multiple Linear Regression
RMSE	Root Mean Square Error
RF	Random Forest
R^2^	Coefficient of Determination
SARIMA	Seasonal Autoregressive Integrated Moving Average
SD	Standard Deviation
SDG	Sustainable Development Goal
SE	Standard Error
SVR	Support Vector Regression
VAR	Vector Autoregression
VIF	Variance Inflation Factor

## References

[1] Halpern B.S., Frazier M., Verstaen J., Rayner P.-E., Clawson G., Blanchard J.L., Cottrell R.S., Froehlich H.E., Gephart J.A., Jacobsen N.S. (2022). The environmental footprint of global food production. Nat. Sustain..

[2] Ignaciuk A., Ilicic J., Asprooth L., Sitko N.J., Bernard A., Maggio G., Tubiello F.N., Mueller M. (2021). Progress Towards Sustainable Agriculture–Drivers of Change.

[3] Kline K.L., Msangi S., Dale V.H., Woods J., Souza G.M., Osseweijer P., Clancy J.S., Hilbert J.A., Johnson F.X., McDonnell P.C. (2017). Reconciling food security and bioenergy: Priorities for action. GCB Bioenergy.

[4] Jevtic M., Bouland C. (2022). Food Production as a challenge for One Health Approach and Public Health. Eur. J. Public Health.

[5] (2022). FAO_Greenhouse_Gas_Emissions. Food Systems—Global, Regional and Country Trends, 2000–2020. FAOSTAT Anal. Br. Ser..

[6] Aktürk E., Gültekin S. (2025). The impact of food production on ecological footprint in Turkey: An analysis across agriculture, livestock, and aquaculture. Environ. Dev. Sustain..

[7] Erdogan S. (2022). Investigating the effects of food production on sustainable development: The case of the upper middle-income countries. Sustain. Dev..

[8] Almohaimeed F.A., Abouelnour K.A. (2025). The role of food processing in sustaining food security indicators in the Kingdom of Saudi Arabia. Economies.

[9] İslamoğlu H. (2017). The politics of agricultural production in Turkey. Neoliberal Turkey and Its Discontents: Economic Policy and the Environment Under Erdogan.

[10] Gürbüzer G.B., Çiftci F. (2025). Projections of agricultural product exports and their impacts on rural development. Tekirdağ Ziraat Fakültesi Derg..

[11] Koç A.A., Bayaner A., Koç G. (2024). Agri-food policy trends and state of sustainable food system in Türkiye. New Medit..

[12] Yontar E., Ersöz S. (2020). Investigation of food supply chain sustainability performance for Turkey’s food sector. Front. Sustain. Food Syst..

[13] Box G.E., Jenkins G.M., Reinsel G.C., Ljung G.M. (2015). Time Series Analysis: Forecasting and Control.

[14] Hyndman R.J., Athanasopoulos G. (2018). Forecasting: Principles and Practice.

[15] Garba M.K., Akanni S.B., Kareem K.Y., Yusuf A.A., Jabaru S.O., Abolarin J.S., Amoyedo F.E., Ekundayo S.O. (2023). Modelling of Post-COVID-19 Food Production Index in Nigeria using Box-Jenkins Methodology. Sule Lamido Univ. J. Sci. Technol..

[16] Kerdprasop K., Chuaybamroong P., Kerdprasop N. (2022). Food Production Forecasting with Time Series and Ensemble Modeling Methods. Proceedings of the 2022 5th Artificial Intelligence and Cloud Computing Conference.

[17] Nosratabadi S., Ardabili S., Lakner Z., Mako C., Mosavi A. (2021). Prediction of food production using machine learning algorithms of multilayer perceptron and ANFIS. Agriculture.

[18] Aworka R., Cedric L.S., Adoni W.Y.H., Zoueu J.T., Mutombo F.K., Kimpolo C.L.M., Nahhal T., Krichen M. (2022). Agricultural decision system based on advanced machine learning models for yield prediction: Case of East African countries. Smart Agric. Technol..

[19] Badshah A., Alkazemi B.Y., Din F., Zamli K.Z., Haris M. (2024). Crop classification and yield prediction using robust machine learning models for agricultural sustainability. IEEE Access.

[20] Jabed M.A., Murad M.A.A. (2024). Crop yield prediction in agriculture: A comprehensive review of machine learning and deep learning approaches, with insights for future research and sustainability. Heliyon.

[21] Dhal S.B., Kar D. (2024). Transforming agricultural productivity with AI-driven forecasting: Innovations in food security and supply chain optimization. Forecasting.

[22] Herteux J., Raeth C., Martini G., Baha A., Koupparis K., Lauzana I., Piovani D. (2024). Forecasting trends in food security with real time data. Commun. Earth Environ..

[23] Mishra H., Mishra R., Olayinka O.T., Ghaib A.A., Raza M.Y., Maroof K. (2025). The Role of Digital Agriculture in Mitigating Climate Change and Ensuring Food Security: An Overview. Climate Resilient and Sustainable Agriculture: Volume 2: Social and Transformative Strategies.

[24] Ali Z., Muhammad A., Lee N., Waqar M., Lee S.W. (2025). Artificial intelligence for sustainable agriculture: A comprehensive review of AI-driven technologies in crop production. Sustainability.

[25] Wolpert D.H., Macready W.G. (1997). No free lunch theorems for optimization. IEEE Trans. Evol. Comput..

[26] Kayakuş M., Akkaya C. (2026). Predicting Turkey’s Sustainable Development Performance Using Machine Learning. Sustain. Dev..

[27] World Bank Open Data (2026). Türkiye Data.

[28] Van der Ploeg J.D., Cloke P., Marsden T., Mooney P.H. (2006). Agricultural production in crisis. Handbook of Rural Studies.

[29] Sarker I.H. (2021). Machine learning: Algorithms, real-world applications and research directions. SN Comput. Sci..

[30] Pan I., Mason L.R., Matar O.K. (2022). Data-centric Engineering: Integrating simulation, machine learning and statistics. Challenges and opportunities. Chem. Eng. Sci..

[31] Kyriazos T., Poga M. (2024). Application of machine learning models in social sciences: Managing nonlinear relationships. Encyclopedia.

[32] Etemadi S., Khashei M. (2021). Etemadi multiple linear regression. Measurement.

[33] Valentini M., dos Santos G.B., Muller Vieira B. (2021). Multiple linear regression analysis (MLR) applied for modeling a new WQI equation for monitoring the water quality of Mirim Lagoon, in the state of Rio Grande do Sul—Brazil. SN Appl. Sci..

[34] Hill J., Linero A., Murray J. (2020). Bayesian additive regression trees: A review and look forward. Annu. Rev. Stat. Appl..

[35] Van de Schoot R., Depaoli S., King R., Kramer B., Märtens K., Tadesse M.G., Vannucci M., Gelman A., Veen D., Willemsen J. (2021). Bayesian statistics and modelling. Nat. Rev. Methods Prim..

[36] Ma Z., Chen G. (2018). Bayesian methods for dealing with missing data problems. J. Korean Stat. Soc..

[37] Khan S.N., Khan S.U., Aznaoui H., Şahin C.B., Dinler Ö.B. (2023). Generalization of linear and non-linear support vector machine in multiple fields: A review. Comput. Sci. Inf. Technol..

[38] Du K.-L., Jiang B., Lu J., Hua J., Swamy M. (2024). Exploring kernel machines and support vector machines: Principles, techniques, and future directions. Mathematics.

[39] Sagi O., Rokach L. (2018). Ensemble learning: A survey. Wiley Interdiscip. Rev. Data Min. Knowl. Discov..

[40] Zhang Y., Liu J., Shen W. (2022). A review of ensemble learning algorithms used in remote sensing applications. Appl. Sci..

[41] Tyralis H., Papacharalampous G. (2021). Boosting algorithms in energy research: A systematic review. Neural Comput. Appl..

[42] Malashin I., Tynchenko V., Gantimurov A., Nelyub V., Borodulin A. (2025). Boosting-based machine learning applications in polymer science: A review. Polymers.

[43] Saini R. (2025). A review on artificial neural networks for structural analysis. J. Vib. Eng. Technol..

[44] Thakur A., Konde A. (2021). Fundamentals of neural networks. Int. J. Res. Appl. Sci. Eng. Technol..

[45] Jain N., Kumar R. (2022). A review on machine learning & it’s algorithms. Int. J. Soft Comput. Eng..

[46] Suyal M., Goyal P. (2022). A review on analysis of k-nearest neighbor classification machine learning algorithms based on supervised learning. Int. J. Eng. Trends Technol..

[47] Boateng E.Y., Otoo J., Abaye D.A. (2020). Basic tenets of classification algorithms K-nearest-neighbor, support vector machine, random forest and neural network: A review. J. Data Anal. Inf. Process..

[48] Dimitsaki S., Gavriilidis G.I., Dimitriadis V.K., Natsiavas P. (2023). Benchmarking of machine learning classifiers on plasma proteomic for COVID-19 severity prediction through interpretable artificial intelligence. Artif. Intell. Med..

[49] Hodson T.O. (2022). Root mean square error (RMSE) or mean absolute error (MAE): When to use them or not. Geosci. Model Dev..

[50] Hastie T., Tibshirani R., Friedman J. (2009). The Elements of Statistical Learning: Data Mining, Inference, and Prediction.

[51] Goodfellow I. (2016). Deep Learning.

[52] Shalev-Shwartz S., Ben-David S. (2014). Understanding Machine Learning: From Theory to Algorithms.

[53] Kuhn M., Johnson K. (2019). Feature Engineering and Selection: A Practical Approach for Predictive Models.

[54] Makridakis S., Spiliotis E., Assimakopoulos V. (2018). The M4 Competition: Results, findings, conclusion and way forward. Int. J. Forecast..

